# Vaginal microbiota and HPV clearance: A longitudinal study

**DOI:** 10.3389/fonc.2022.955150

**Published:** 2022-10-24

**Authors:** Wenpei Shi, Haiyan Zhu, Lei Yuan, Xiaoyue Chen, Xiaojie Huang, Kai Wang, Zhen Li

**Affiliations:** ^1^ Clinical Research Unit, Shanghai Key Laboratory of Maternal Fetal Medicine, Shanghai Institute of Maternal-Fetal Medicine and Gynecologic Oncology, Shanghai First Maternity and Infant Hospital, School of Medicine, Tongji University, Shanghai, China; ^2^ Department of Gynecology, Shanghai Key Laboratory of Maternal Fetal Medicine, Shanghai Institute of Maternal-Fetal Medicine and Gynecologic Oncology, Shanghai First Maternity and Infant Hospital, School of Medicine, Tongji University, Shanghai, China; ^3^ Clinical and Translational Research Center, Shanghai Key Laboratory of Maternal Fetal Medicine, Shanghai Institute of Maternal-Fetal Medicine and Gynecologic Oncology, Shanghai First Maternity and Infant Hospital, School of Medicine, Tongji University, Shanghai, China

**Keywords:** vaginal microbiota, high-risk human papillomavirus, HPV clearance, longitudinal study, cervical lesions

## Abstract

Although vaginal microbiota (VM) may interact with human papillomavirus (HPV) infection and clearance, longitudinal data remain very limited. We aimed to investigate the association between VM at baseline and the clearance of high-risk HPV (HR-HPV) infection within 12 months. Cervical swabs were collected at diagnosis from 85 patients with HR-HPV infection and histologically confirmed cervical lesions, including cervicitis, low-grade squamous intraepithelial lesion and high-grade squamous intraepithelial lesion. Microbiome analysis was performed using 16S rRNA gene sequencing. Among the 73 women included in the analyses, HPV clearance was observed in 58.9% of the patients within 12 months. No significant difference was observed between the HPV-cleared and HPV-uncleared groups regarding age, disease stage, HPV subtype, VM community state types, and VM diversity (α and β). Women with the depletion of enterococcus ASV_62 and enrichment in *Lactobacillus iners* at baseline were less likely to have HPV clearance at month 12. Further analysis revealed a significant negative association between high abundance of *L. iners* and HPV clearance in patients who received non-operative treatment (OR = 3.94, p = 0.041), but not in those who received operative treatment (OR = 1.86, p = 0.660). Our findings provide new evidence for the potential role of VM in the persistent HR-HPV infections.

## Introduction

Persistent infection with high-risk human papillomavirus (HR-HPV) is the major cause of cervical cancer and its precursor lesion (1–3). Although HPV infection is common worldwide, approximately 80% of the HPV infections are transient and cleared spontaneously within 2 years, and the remaining 20% of cases ensue persistent infection and disease progression. However, the exact factors that determine infection and/or disease that will persist, progress, or spontaneously resolve are incompletely understood.

More recently, the upsurge in research on microbiome has shifted attention from known epidemiological risk factors of HPV infection, including parity, tobacco smoking, and oral contraceptive use to mucosal microbiota (4). Accumulating evidence has revealed that mucosal microbiota plays a critical role in maintaining physiological homeostasis (5). Several recent studies have highlighted the significance of the microbiome in the natural history of various viral infections and cancers (6, 7). The cervicovaginal microbiome is of particular interest in gynecology because it has been well characterized and several specific features have been associated with gynecologic diseases (8–10).

The vaginal microbiota (VM) is commonly categorized into community state types (CSTs), which were first proposed by Ravel et al. (9), and are generally defined as a dominant of a specific *Lactobacillus* spp. or a polymicrobial state with high diversity. Several cross-sectional studies (11–17) have observed distinct characteristics of VM between healthy controls and patients with HPV infection or squamous intraepithelial lesion (SIL). A link between high abundance of some types of *Lactobacillus* (*L. crispatus*, *L. jensenii*, and *L. gasseri*) and low HPV prevalence is generally supported (18). Contrary to the beneficial role of these *Lactobacillus* spp., current evidence with regard to *L. iners*, which was common in patients with HR-HPV infections and high-grade cervical lesions, remains inconsistent. In addition, high-diversity CSTs and specific anaerobes, such as *Sneathia* and *Gardnerella vaginalis*, were also found to be implicated with higher frequency and severity of disease. Nevertheless, the results of previous studies are inconsistent and sometimes contradictory, possibly because of the differences in the genetic background or environmental factors (18, 19). Additionally, it remains unclear whether features of VM influence the clearance or instead promote disease development.

Herein, we conducted a longitudinal study to assess the impact of VM composition at baseline on the clearance of HR-HPV infections at 12 months based on a treatment cohort of 85 Chinese women with a single HR-HPV infection.

## Methods

### Ethics statement

The study was conducted according to the guidelines of the Declaration of Helsinki and approved by the Scientific and Ethical Committee of the Shanghai First Maternity and Infant Hospital affiliated with Tongji University (protocol code: K08-018).

### Study design

Patients with histologically diagnosed cervical disease were enrolled between April 2015 and October 2016 at Shanghai First Maternity and Infant Hospital and then routinely followed up every 6 months. Cervical swab specimens at diagnosis were collected from every woman for HPV testing and microbiome analysis using 16S rRNA gene sequencing after inclusion in the cohort. Detailed description of the participants can be obtained from the original publication (20). All the data were collected after obtaining written informed consent from participants.

Inclusion criteria included the following: biopsy-proven clinical lesions of the cervix, including cervicitis, low-grade SIL (LSIL), and high-grade SIL (HSIL); only infected with one HR-HPV subtype (HPV 16/52/58); follow-up data available; and patients with same diagnostic conditions were treated according to a standardized protocol. Briefly, patients with HSIL received the loop electrosurgical excisional procedure (LEEP), and other patients with a lower pathological grade lesion (cervicitis and LSIL) received conservative management rather than immediate excisional treatment. Patients with a known malignancy disease or current pregnancy were excluded from the study.

### Follow-up and clinical outcome definition

Based on pathological diagnosis and the treatment undergone, patients were divided into the HPV+/LSIL group and the HSIL group. Cervical swabs and cervical biopsies were collected on each follow-up visit for HPV DNA detection and pathological examination. Clinicopathological and follow-up data, including progress notes, clinical laboratory tests, and drugs and pathological reports were captured from the hospital electronic medical record system. All laboratory examinations and inpatient and outpatient electronic medical records were reviewed.

The HPV-cleared group was defined as the presence of HPV at baseline turned negative at follow-up tests and no further positive HPV test and cytological or histological abnormality reports. The HPV-uncleared group was defined as persistent same-type HPV infection or pathological progress within a 1-year follow-up.

### HPV gene type

HPV testing at each follow-up visit was performed using the HPV GenoArray test kit as previously reported (20). The absence of HPV DNA contamination was confirmed by HPV L1 and the internal control of the human a-globin in each reaction.

### 16S rRNA V4–V5 amplicon sequencing

Microbial genomic DNA was extracted from cervical swab samples, collected from the ectocervix and endocervix of the uterus of every woman by baseline cervical scrapings, using the FastDNA Spin Extraction Kit (MP Biomedicals, Santa Ana, CA, USA). Then, a nested PCR protocol was employed to amplify the 16S hypervariable region V4–V5 using the 16S universal primers: 515F 5’-GTGCCAGCMGCCGCGGTAA-3’ and 907R: 5’-CCGTCAATTCMTTTRAGTTT-3’. The dual-indexed amplicons were pooled according to the manufacturer’s instructions (Illumina, Inc., San Diego, CA, USA) and sequenced on the Illumina MiSeq platform to produce 2 × 300 bp paired-end reads as described previously.

### Bioinformatic analysis

The raw demultiplexed sequences were firstly trimmed off primers from the paired-end reads, and a preliminary quality trimming was then conducted with Cutadapt v2.10 with the setting of discard untrimmed sequences, a minimum *q*-value of 20, and a maximum *N* base of zero. Thereafter, the processed reads were subjected to quality trimming, denoising, merging, and chimera removal to generate amplicon sequence variants (ASVs) using DADA2 (21). In this step, the paired-end reads were trimmed to keep high-quality reads with a *q*-value of >20 (maxN = 0, truncQ = 2), and those with more than two or five expected errors [maxEE = c(2,5)] or derived from PhiX (rm.phix = TRUE) were discarded. After DADA2 denoising, the paired-end reads were merged with at least a 12-bp overlap. Chimera checking was conducted on the merged reads, and the recovered ASVs were summarized and used to generate the sequence table for the sequencing run. All ASVs were numbered in order.

### Sequencing annotation

RESCRIPt (22) was used to compile trained naïve Bayes classifiers using the SILVA database (v132) (23) and the STIRRUPS vaginal microbiome-specific database (24). As *Lactobacillus* spp. are essential for further analysis in VM studies, the classification of the ASVs annotated as *Lactobacillus* was improved by manually BLAST searching them in the National Center for Biotechnology Information database; a maximum of one mismatch was allowed from the alignment. If ASVs could not be annotated at the species level, they were then reannotated as “genus-level ASVs order.”

### Diversity analysis

The ASVs table was filtered to exclude sequences annotated as chloroplasts, plastids, or non-bacterial ASVs. Low-abundance ASVs present in only one sample and with a relative abundance lower than 0.01% across all ASVs were also filtered. To reduce the sampling heterogeneity, the ASV table was rarefied to the same reads per sample 100 times using a q2-repeat-rarefy plugin (25) on the QIIME2 platform before conducting diversity analysis. The within-sample (α) diversity was calculated using Chao1 and Shannon indexes based on species richness and species frequencies. The Bray–Curtis distance between samples (β diversity) was used to evaluate differences in species complexity. Both α-diversity indexes and β-diversity distance were calculated using QIIME2 (26).

### Clustering into community state types

Hierarchical clustering into CSTs based on VM composition and abundance was conducted according to the methods described by DiGiulio et al. (27). Specifically, the Bray–Curtis distance matrix between all samples was denoised by extracting the most significant principal coordinates analysis (PCoA) eigenvectors. Then, the partitioning around medoids algorithm (pam) was applied to PCoA distances. The number of clusters was determined from the gap statistic. According to this algorithm, VM composition was classified into five groups at the ASV level and two groups at the genus level.

### Statistical analysis

Normality tests for continuous data were assessed by Shapiro–Wilk tests. Chi-square tests, Fisher exact tests, *t*-tests, and Wilcoxon rank-sum tests were used for two-group comparisons as appropriate. For further analysis, numerical variables such as α-diversity indexes were categorized by 75th quartile scores.

PCoA analysis was performed to interrogate the robustness of group-wise clustering. Comparisons of group-wise β diversity (Bray–Curtis distance matrix) were assessed by permutational multivariable ANOVA using the Adonis function in the vegan package (28). Logistic regression models were performed to adjust for known confounders (age, HPV subtype) and calculated adjusted odds ratios (aOR) to evaluate the relationship between CSTs and clinical outcomes. Analyses of differential taxa (species level) abundance of samples according to disease outcome were performed using a negative binomial generalized linear model in the R package DESeq2 (29) with a multifactor design. An adjusted p-value of <0.05 and an estimated fold change of >2 were considered significantly differentially abundant between groups.

P-value was adjusted for multiple tests using the Benjamini and Hochberg method. A p-value of <0.05 was considered statistically significant. Statistical analyses were performed with R statistical programming (R version 3.6.1).

## Results

### Characteristics of participants in the study cohort and follow-up

**Figure 1:**
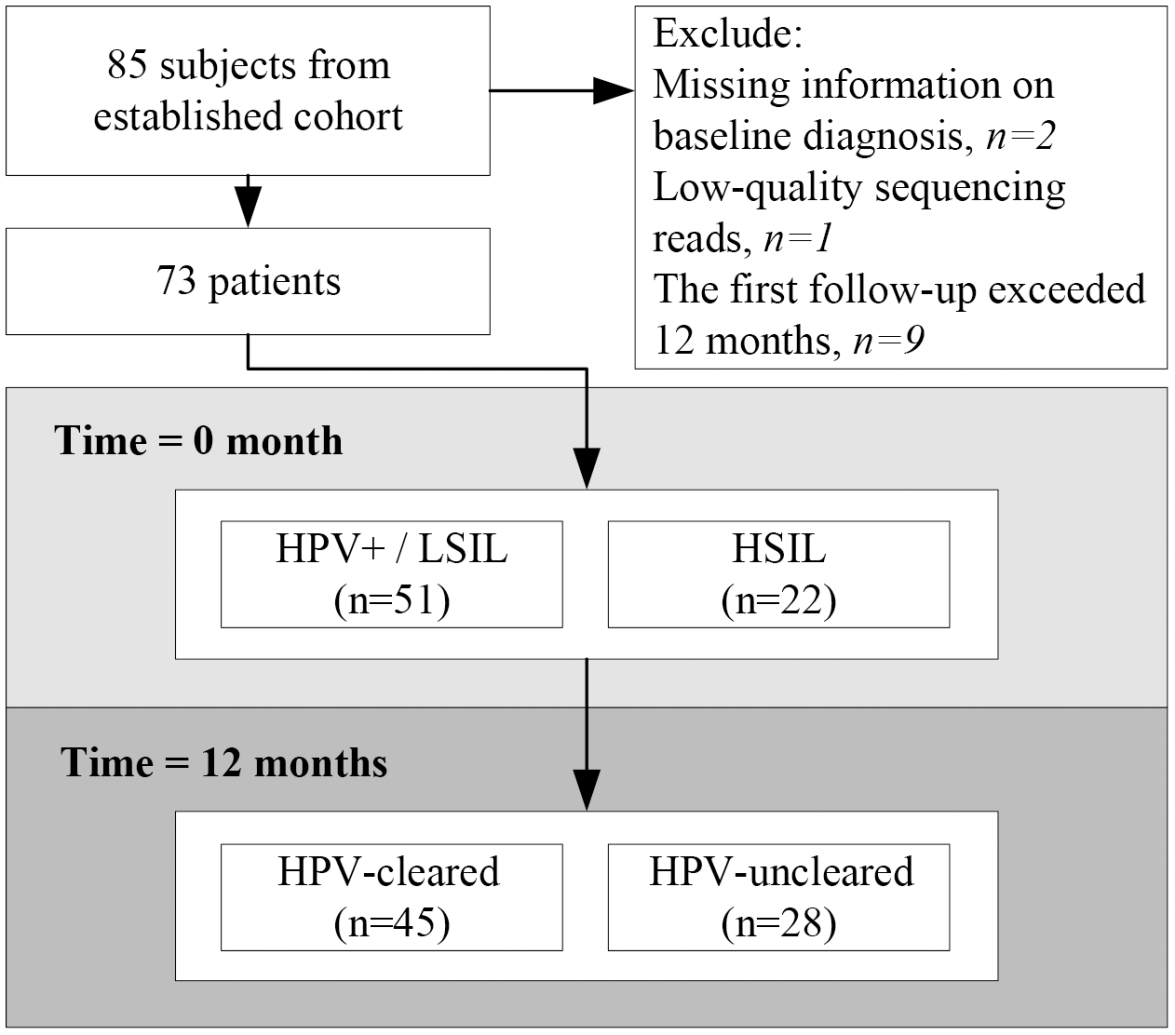
Flow chart of the study design. Seventy-three patients with histologically confirmed cervical lesion at baseline entered this study. Follow-up cytology and human papillomavirus (HPV) tests were performed every 6 months to determine whether subjects had cleared HPV infections or not at 12 months.

Eighty-five patients with a single HR-HPV infection and histologically confirmed cervical disease were enrolled in this study. Patients with incomplete clinical information (n = 2), low-quality sequence data (n = 1), or time to first follow-up visit over 1 year (n = 9) were excluded. Overall, 73 individuals were included in the analysis (**Figure 1**). Patient characteristics are detailed in **Table 1**. The mean age was 40.1 ± 11.5 years old (median = 36, range: 24–68). The majority of subjects (69.9%) had low-grade cervical lesions. All participants were infected with a single HPV subtype, of whom 37.0% were infected with HPV52, followed by 35.6% with HPV16 and 27.4% with HPV58. Sixteen (21.9%) patients had taken recombinant human interferon α-2b, and five (6.8%) had taken *Lactobacillus* capsule. No immunomodulatory medication usage was reported. At 12 months, 61.6% (45/73) of patients had cleared HPV infection, defined as the “HPV-cleared” group, and the remaining 38.4% (28/73) were classified as the “HPV-uncleared” group. The clearance rate was 54.9% among HPV+/LSIL patients and 77.3% among HSIL patients. This difference of clearance rate between the two groups was not statistically significant (p = 0.123). No differences in HPV clearance by HPV subtype were noted (HPV16, 65.4%; HPV52, 59.3%; HPV58, 60.0%; p = 0.886). Pairwise comparisons are shown in **Supplementary Table S1**.

**Table 1:** Clinical characteristics and outcomes of participants in the study.

Characteristics	HPV-cleared	HPV-uncleared	p-value	Total
	(N = 45)	(N = 28)		(N = 73)
**Age at diagnosis, years**			0.180	
Median [range]	35.0 [24.0–68.0]	36.5 [27.0–65.0]		40.1 (11.5)
**Diagnosis at baseline**			0.123	
HPV+/LSIL	28 (62.2%)	23 (82.1%)		51 (69.9%)
HSIL	17 (37.8%)	05 (17.9%)		22 (30.1%)
**HPV status**			0.886	
HPV16 positive	17 (37.8%)	09 (32.1%)		26 (35.6%)
HPV52 positive	16 (35.6%)	11 (39.3%)		27 (37.0%)
HPV58 positive	12 (26.7%)	08 (28.6%)		20 (27.4%)
**Drug**				
**Recombinant human interferon α-2b**			1.000	
No	35 (77.8%)	22 (78.6%)		57 (78.1%)
Yes	10 (22.2%)	06 (21.4%)		16 (21.9%)
***Lactobacillus* capsule**			1.000	
No	42 (93.3%)	26 (92.9%)		68 (93.2%)
Yes	3 (6.7%)	02 (7.1%)		5 (6.8%)
**CSTs**			0.216	
I	6 (13.3%)	04 (14.3%)		10 (13.7%)
II	15 (33.3%)	07 (25.0%)		22 (30.1%)
III	6 (13.3%)	09 (32.1%)		15 (20.5%)
IV	10 (22.2%)	02 (7.1%)		12 (16.4%)
V	8 (17.8%)	06 (21.4%)		14 (19.2%)
**CST_genus**			1.00	
1	28 (62.2%)	18 (64.3%)		46 (63.0%)
2	17 (37.8%)	10 (35.7%)		27 (37.0%)
**Chao1**			0.074	
Median [min, max]	36.5 [8.00, 134]	27.3 [6.00, 99.1]		29.0 [6.00, 134]
**Pielou_evenness**			0.863	
Mean (SD)	0.387 (0.166)	0.39 (0.164)		0.390 (0.164)
**Observed_features**			0.073	
Median [min, max]	32.0 [8.00, 131]	26.0 [6.00, 98.0]		
**Shannon**			0.664	
Median [min, max]	1.56 [0.143, 4.54]	1.77 [0.327, 3.34]		
**Simpson**			0.987	
Median [Min, max]	0.55 [0.028, 0.928]	0.61 [0.0850, 0.822]		

SD, standard deviation; HPV, human papillomavirus; CSTs, community state types; LSIL, low-grade squamous intraepithelial lesion; HSIL, high-grade squamous intraepithelial lesion. HSIL patients received surgical resection, and LSIL/HPV+ patients underwent non-surgical treatment.

Comparisons between groups were made using chi-square test, t-test, Wilcoxon rank-sum test, and Fisher exact test as appropriate. Age at diagnosis, Chao1, observed features, Shannon, and Simpson were non-normal distribution tested by Shapiro–Wilk test.

### Characteristics of baseline vaginal microbiota composition

A total of 2,785,739 high-quality sequences were obtained from 73 samples, with an average of 38,160 reads per sample. Following the removal of rare frequency (singletons and <0.1% total reads), nonbacterial, unclassified, mitochondrial, and chloroplast ASVs, 530 ASVs were finally generated, and then all samples were rarefied to 8,556 reads 100 times to calculate diversity indexes.

**Figure 2:**
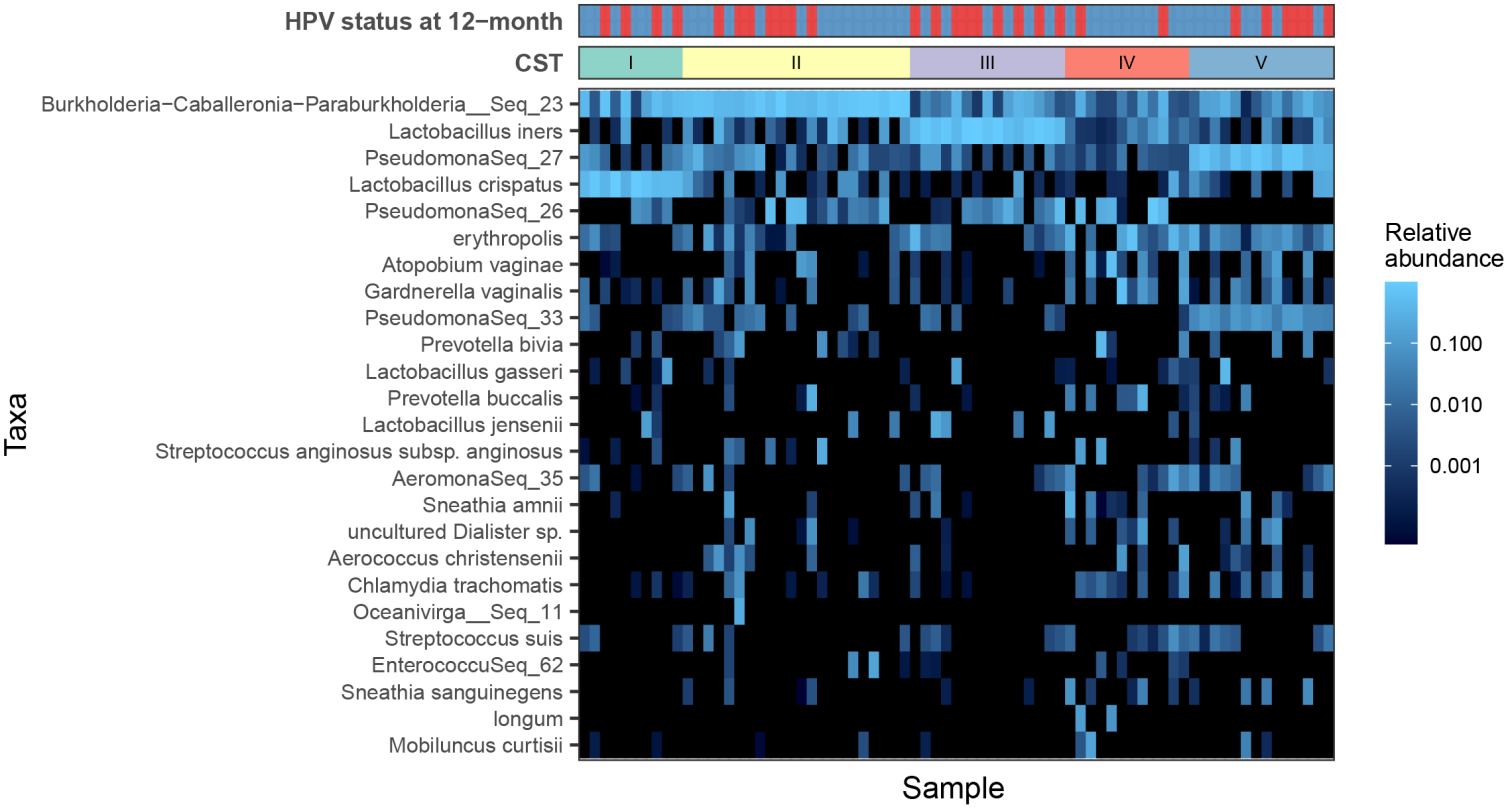
Heat map of the fractional abundance of the 25 most abundant amplicon sequence variants (ASVs) in the vaginal communities of all subjects. Clustering on the abundance profiles of individual samples using the partitioning around medoids algorithm identifies five community state types. Human papillomavirus (HPV) infection outcomes are indicated by the bar at the top: HPV negative (blue) and HPV positive (red).

To explore CSTs and reduce dimensionality, a hierarchical clustering analysis was performed based on ASV-level data, and five major groups were identified (**Figure 2** and **Figures S1A–E**): CST I (10/73, 13.7%) was classified as *L. crispatus* dominated, CST III was dominated by *L. iners* (15/73, 20.5%), and CST IV (12/73, 16.4%) was typified by a highly diverse microbiome rather than containing a major group.

A similar genus-level analysis demonstrated that all the samples were mainly separated into two groups, the *Lactobacillus*-dominated group (CST1, 63.0%) and the non–*Lactobacillus*-dominated group (CST2, 37.0%) (**Figures S1F, G**). Alpha diversity analysis of the microbiota profile based on Shannon and Chao1 diversity showed that patients with HPV-16 infection or non–*Lactobacillus*-dominated CST had higher VM diversity (**Tables S2** and **S3**). PCoA plot of Bray–Curtis distances showed a clear separation of microbial composition between different CSTs (**Figure S2**). Although no significant correlation was observed between VM composition (measured by Bray–Curtis) and clinical variables, including HPV subtype, age, and disease stage (**Tables S4** and **S5**).

### Overall vaginal microbiota diversity and HPV clearance

**Figure 3:**
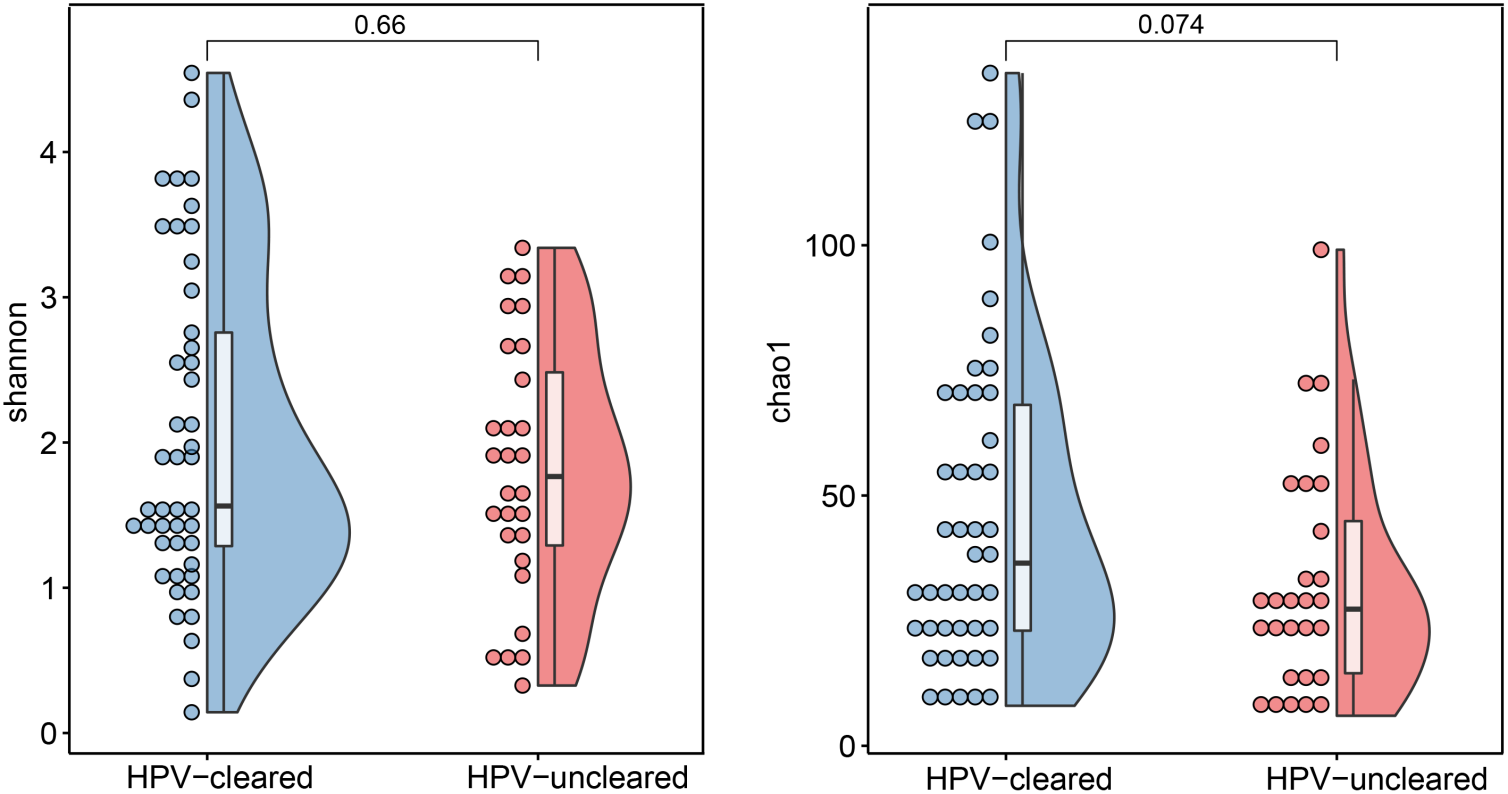
Microbial α-diversity analysis based on Shannon and Chao1 index in the HPV-cleared and HPV-uncleared groups. Statistical significance between the groups was tested by Wilcoxon rank sum tests. HPV, human papillomavirus.

**Figure 4:**
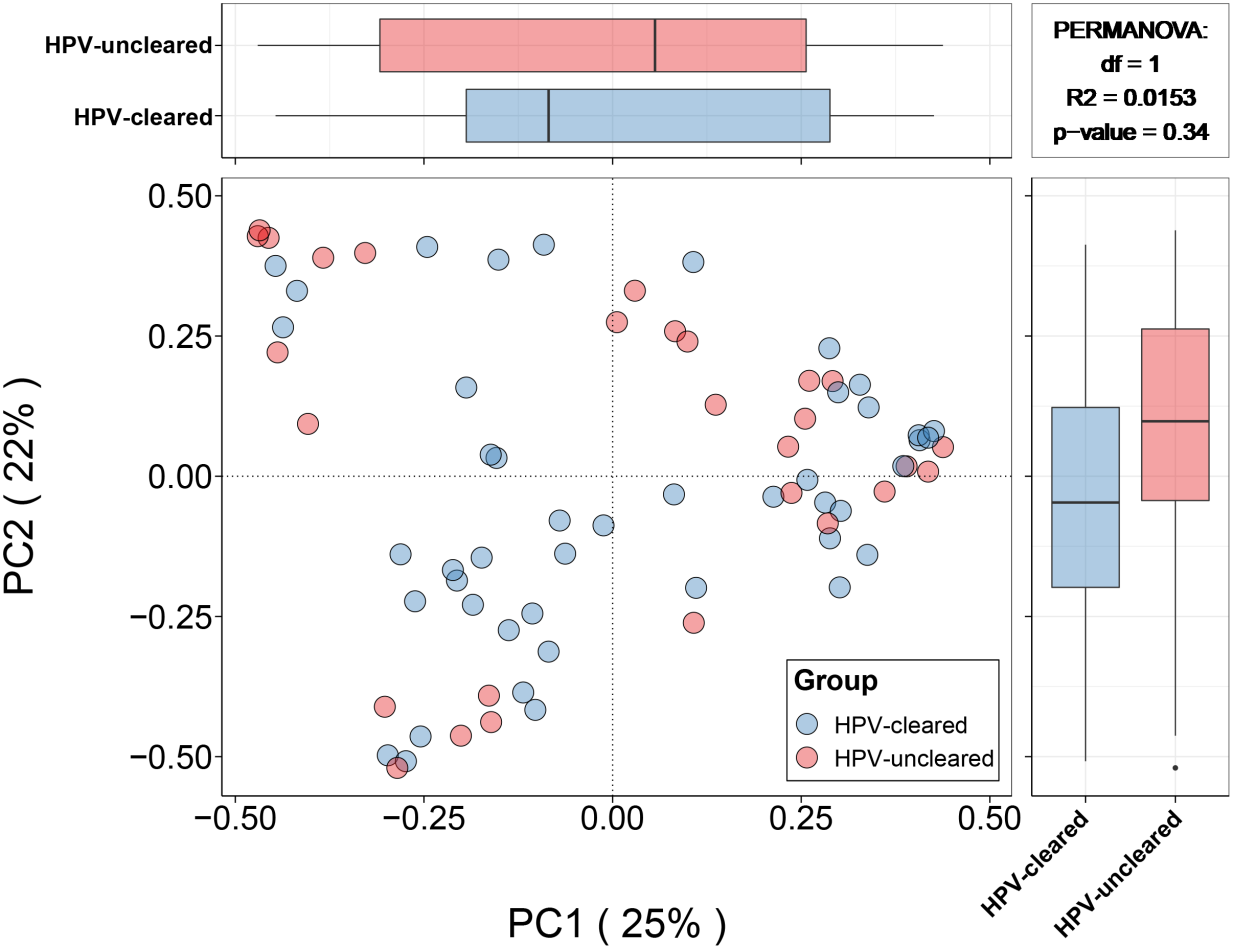
Principal coordinates analysis (for principal coordinates PCo1 and PCo2) plots with Bray–Curtis distance showing the difference in microbial community composition between the HPV-cleared and HPV-uncleared groups. Statistical significance between the groups was tested by permutational multivariable ANOVA. HPV, human papillomavirus.

At 12 months, clearance rates appeared to be higher in HSIL patients than in patients with HPV+/LSIL, bordering on significance (77.3 vs. 54.9%, p = 0.123). Both α-diversity and β-diversity analyses showed no significant difference between the HPV-cleared and HPV-uncleared groups (**Figure 3**). Similarly, Bray–Curtis distance showed no clear separation among samples from different HPV treatment outcome groups (**Figure 4**). In addition, these data were supported by the fact that no significant difference was found between VM features and HPV outcomes in univariate and multivariate logistic regression analyses (**Table 2**). Further stratified analysis by HPV subtype and disease stage did not reveal any significant differences between the two groups (**Tables S6** and **S7**).

**Table 2:** Association between VM and HPV clearance in a 12-month follow-up.

Characteristics	Univariable model		Multivariable model 1		Multivariable model 2		Multivariable model 3		Multivariable model 4	
	OR (95% CI)	p value	aOR (95% CI)	p value	aOR (95% CI)	p value	aOR (95% CI)	p value	aOR (95% CI)	p value
**Age**										
<50 year	Ref.		Ref.		Ref.		Ref.		Ref.	
≥50 year	1.69 (0.96–2.98)	0.070	3.38 (0.89–12.8)	0.073	2.4 (0.71–8.08)	0.157	3.78 (0.97–14.69)	0.055	2.46 (0.73–8.32)	0.147
**HPV type**										
16	Ref.		Ref.		Ref.		Ref.		Ref.	
52	1.3 (0.43–3.96)	0.646	1.2 (0.33–4.38)	0.782	0.93 (0.28–3.09)	0.901	1.31 (0.36–4.82)	0.683	0.97 (0.29–3.29)	0.963
58	1.26 (0.38–4.20)	0.708	0.99 (0.25–3.94)	0.987	0.78 (0.21–2.96)	0.720	1.09 (0.27–4.43)	0.908	0.81 (0.21–3.07)	0.755
**Diagnosis**										
HPV+/LSIL	Ref.				Ref.		Ref.		Ref.	
HSIL	0.5 (0.22–1.15)	0.105	0.38 (0.1–1.4)	0.146	0.36 (0.11–1.24)	0.106	0.34 (0.09–1.28)	0.110	0.35 (0.1–1.2)	0.096
**Shannon**										
Low	Ref.		Ref.		Ref.		Ref.		Ref.	
High	0.75 (0.25–2.30)	0.614	1.14 (0.27–4.76)	0.858	0.84 (0.22–3.16)	0.794	0.87 (0.19–3.95)	0.858	0.7 (0.2–2.49)	0.577
**CSTs**										
I	Ref.		Ref.				Ref.			
II	0.70 (0.15–3.30)	0.652	0.38 (0.07–2.22)	0.284			0.38 (0.06–2.27)	0.288		
III	2.25 (0.44–11.52)	0.330	1.53 (0.27–8.55)	0.630			1.91 (0.31–11.62)	0.484		
IV	0.30 (0.04–2.16)	0.232	0.13 (0.01–1.25)	0.077			0.13 (0.01–1.27)	0.079		
V	1.12 (0.22–5.86)	0.889	0.6 (0.09–4.17)	0.609			0.84 (0.11–6.39)	0.863		
**CST_genus**										
I	Ref.				Ref.				Ref.	
II	0.92 (0.34–2.44)	0.859			0.75 (0.22–2.59)	0.649			0.73 (0.24–2.28)	0.594
**Recombinant human interferon α-2b**										
No	Ref.						Ref.		Ref.	
Yes	0.95 (0.3–3)	0.936					2.47 (0.57–10.78)	0.229	1.38 (0.39–4.91)	0.615
***Lactobacillus* capsule**										
No	Ref.									
Yes	1.08 (0.17–6.88)	0.938								

VM, vaginal microbiota; HPV, human papillomavirus; LSIL, low-grade squamous intraepithelial lesion; HSIL, high-grade squamous intraepithelial lesion; CSTs, community state types; aOR, adjusted odds ratios. HSIL patients received surgical resection, and LSIL/HPV+ patients underwent non-surgical treatment.

### Certain bacterial species showed correlation to human papillomavirus treatment outcome

Analysis of bacterial taxonomic categories of the microbiome associated with HPV-uncleared versus HPV-cleared was performed using DESeq2, which built a generalized linear model to validate the abundance of each taxon with adjustments for disease stage, age group, and HPV subtype. DESeq2 results showed that abundances of the two species (one specie enriched and one specie depleted in HPV-cleared group) presented a significant difference between two groups. The abundance of *Enterococcus:ASV_62* (|log-fold change| = 8.84, *q* < 0.001) was significantly more prevalent among HPV-cleared patients. Interestingly, patients with higher abundance of *L. iners* at baseline were more likely to fail to clear HPV infection at 12 months (|log-fold change| = 4.16, *q* < 0.001).

**Figure 5:**
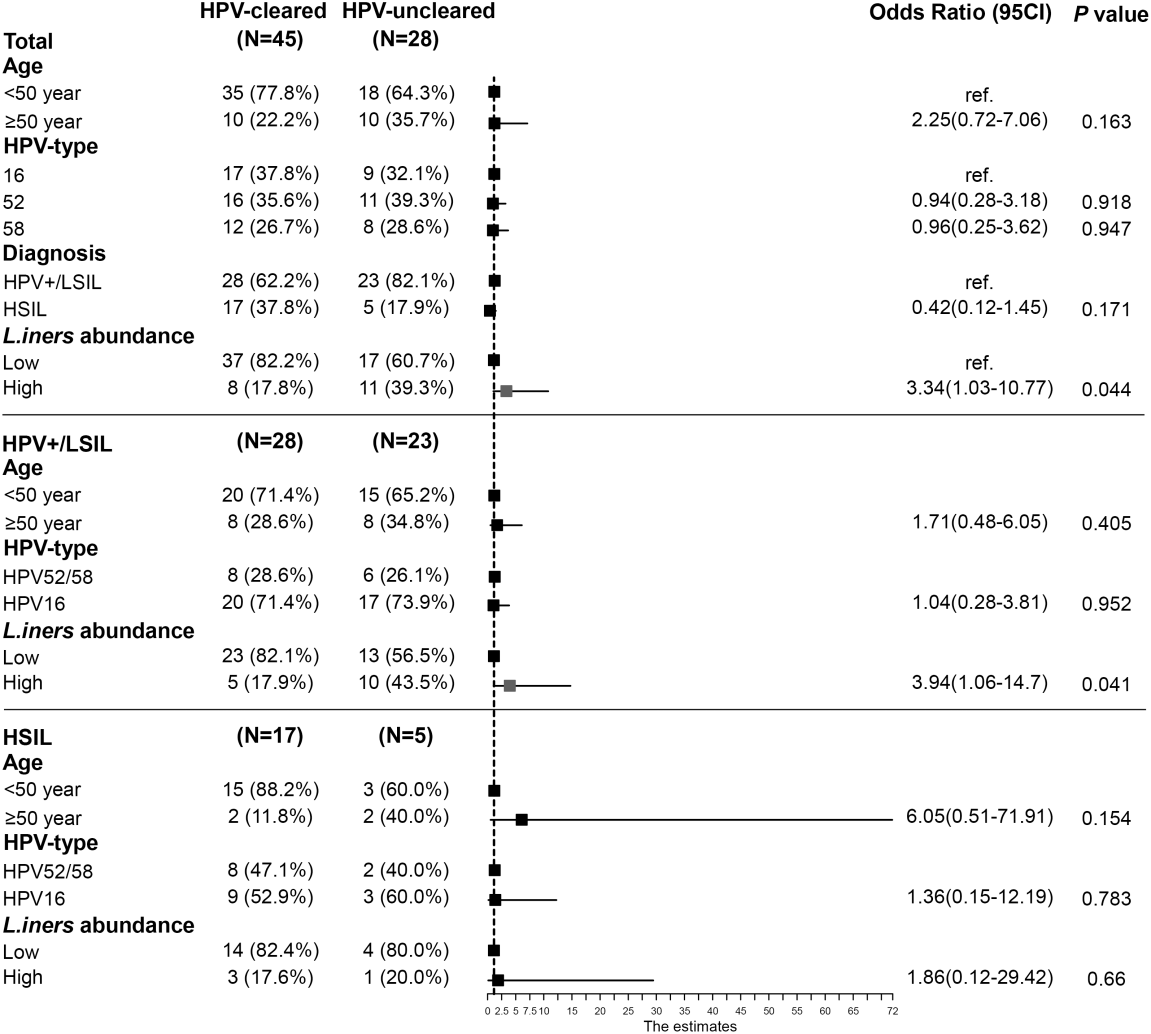
Association between *L. iners* abundance and HPV clearance in a 12-month follow-up. Forest plots were shown using data from multivariable logistic regression in total and according to disease status. HSIL patients received surgical resection, and LSIL/HPV+ patients underwent a surgical treatment. The abundance of *L. iners* was categorized by 75th quartile scores. HPV, human papillomavirus; LSIL, low-grade squamous intraepithelial lesion; HSIL, high-grade squamous intraepithelial lesion.

To assess the robustness of the negative correlation between *L. iners* abundance and HPV clearance, a multivariable logistic regression model and stratified analyses were performed to assess the effect of *L. iners* abundance on HPV outcome status. The multivariable analysis revealed a significant risk effect of higher *L. iners* abundance (aOR = 3.34, 95% CI: 1.03–10.77) on HPV clearance (**Figure 5**). Further stratification logistic analysis showed that this association was only observed in patients with HPV+/LSIL (aOR = 3.94, 95% CI: 1.06–14.70), but not in those with HSIL (aOR = 1.86, 95% CI: 0.12–29.42) (**Figure 5**).

## Discussion

To our knowledge, this longitudinal study reported for the first time that the relative abundance of specific cervicovaginal bacteria, *L. iners*, rather than the overall diversity of VM, negatively associated with the clearance of HR-HPV at month 12 after diagnosis among patients who received non-operative treatment. Our findings provide new evidence that VM may influence the clearance of HR-HPV infections and suggested a new potential therapy target that merits further investigation.

After adjustment for potential confounders, we observed the association of the enrichment of *L. iners* and depletion of *Enterococcus ASV_62* with the clearance of HR-HPV. Possibly because of the relatively limited sample size, a non-significant similar trend was seen when comparing to the CST I (*L. crispatus* dominated) state, that patients with CST III (*L. iners* dominated) state at baseline were associated with a lower rate of HPV clearance. Previously, the possible link between *L. iners* and persistent HPV infection has been reported in several cross-sectional studies (11, 17, 30, 31). A recent meta-analysis showed that VM dominated by *L. iners*, compared with VM dominated by *L. crispatus* was associated with a two- to threefold higher risk of HR-HPV infection and dysplasia (32). Although Usyk et al. (33) found that *L. iners* was the most positively associated taxon with clearance at 12 months among young adults form Costa Rica (33). The discrepancies in the findings may be related to different inclusion criteria and race differences across studies. By including patients with a single-type HR-HPV infection, this study reduced possible confounding factors related to interactions within different types of HR-HPV.

Compared to other *Lactobacillus* spp. frequently identified, such as *L. crispatus*, which is usually considered as a biomarker of healthy vaginal microenvironment, current studies support an ambiguous role for *L. iners* in the vaginal niche (34, 35). *L. iners* has a relatively small genome size compared with other *Lactobacillus* spp. and has been reported as a dominant species in the transitional type of the VM (CST III) or during menses or episodes of bacterial vaginosis, suggesting that this species is very flexible and has a remarkable ability to adapt to the fluctuating vaginal environment (34). The genome of *L. iners* also encodes a number of genes, suggesting that it could may be an opportunistic pathogen, of which inerolysin (a potential cholesterol-dependent cytolysin) is well documented (36). Besides, a study examining cytokine profiles in pregnancy women showed a positive association between *L. iners* and proinflammatory cytokines in vaginal fluid (37). However, the available literature is insufficient to classify *L. iners* as a beneficial or detrimental bacterium. Because *L. iners*–dominated CST III is frequently reported as one of the most common CSTs among Asian reproductive-age women, a more detailed approach to explore the causal relationship between *L. iners* and HPV infection clearance is warranted in future vaginal microbiome studies.

Further stratified analysis according to disease status or treatment revealed that the negative correlation between the abundance of *L. iners* and HPV clearance was only observed in HPV+/LSIL patients who received non-operative treatment. One possible reason is that, compared with non-surgical management such as anti-inflammatory or antiviral treatment, the impact of resection of the lesion on HPV clearance is immediate (38). Recently, Mitra et al. (39) found that the surgical excision for HSIL and HPV infection did not alter VM composition, which suggested that the virus is not the driver of VB alternations. In this study, women with histologically confirmed HSIL were immediately treated surgically according to clinical guidelines, whereas conservative management with regular follow-up was recommended for HPV+/LSIL patients. Previous studies (40, 41) reported that the HR-HPV clearance can reach 79.2%–97.8% (40) at 12 months after LEEP. In this study, 77.3% of HSIL patients and 54.9% of HPV+/LSIL patients were cleared of HPV infection within 12 months, which is comparable to other similar studies (41–43). It is likely that the effect of surgical treatment can mask the correlation between *L. iners* and HPV outcome in HSIL patients. These findings provide clues for future research regarding the potential targeting populations regarding the association between VM and HPV persistence.

The study has the following limitations. First, the sample size was relatively limited; thus, the negative findings need to be interpreted with caution because of potential insufficient statistical power. Considering the subtype of HPV could be a confounder factor affecting the association between VM and HPV clearance, this study enrolled patients with one of three most prevalent HR-HPV types (HPV16, HPV52, or HPV58). These were the top three subtypes with the highest HPV infection rates according to our previous study (20) and epidemiological surveys in mainland China (44, 45). Further studies with a larger sample size and more types of HPV infection would allow more detailed analyses by HPV subtypes. Second, as cigarette smoking is rare among Chinese women, we were not able to investigate the effect of smoking as a potential confounder. Reported by the Shanghai municipal center for disease control and prevention, the smoking prevalence among female was 1.03% at 2016 (46). The smoking status is not likely to change the reported associations in this study. Third, a single sampling at baseline cannot establish a definitive causal link between *L. iners* and clearance of HPV infection. Further prospective human studies with repeated sampling and follow-up time longer than 24 months are needed to better clarify the association between VM and persistent HPV infection.

In conclusion, this study among Chinese women suggested that the relative abundance of *L. iners* at diagnosis, rather than overall bacterial diversity, was negatively correlated with HPV clearance over 12 months, particularly in patients who received non-operative treatment. Our findings provide new evidence for the potential role of VM in the persistent HR-HPV infections. Further studies are needed to clarify the mechanisms by which *L. iners* promotes persistent HPV infection or lesion progression.

## Data availability statement

The raw sequence data reported in this paper have been deposited in the Genome Sequence Archive (Genomics, Proteomics & Bioinformatics 2021) in National Genomics Data Center (Nucleic Acids Res 2021), China National Center for Bioinformation / Beijing Institute of Genomics, Chinese Academy of Sciences (GSA: HRA003171) that are publicly accessible at https://ngdc.cncb.ac.cn/gsa.

## Ethics statement

This study was reviewed and approved by Scientific and Ethical Committee of the Shanghai First Maternity and Infant Hospital affiliated with Tongji University. The patients/participants provided their written informed consent toparticipate in this study.

## Author contributions

Conceptualization: WS, HZ, ZL and KW. Methodology: WS and XH. Software: WS and LY. Validation: HZ and XC. Formal analysis: WS and HZ. Investigation, XH, LY and WS. Resources, LY, XH and KW. Data curation: XC and HZ. Writing—original draft preparation: WS, HZ and ZL. Writing—review and editing: WS, HZ, LY, XC, XH, KW and ZL. Visualization: W.S. Supervision: HZ and ZL. Project administration: KW and ZL. All authors contributed to the article and approved the submitted version.

## Funding

This study was supported by grants from the Shanghai First Maternity and Infant Hospital Science Project (WS, 2020A20) and Clinical Research Plan of SHDC (ZL, SHDC2022CRS050). The granters played no role in study design, development, data collection, or generation of this manuscript. There is no other financial support.

## Acknowledgments

We thank all the participating staff for their involvement with this project.

## Conflict of interest

The authors declare that the research was conducted in the absence of any commercial or financial relationships that could be construed as a potential conflict of interest.

## Publisher’s note

All claims expressed in this article are solely those of the authors and do not necessarily represent those of their affiliated organizations, or those of the publisher, the editors and the reviewers. Any product that may be evaluated in this article, or claim that may be made by its manufacturer, is not guaranteed or endorsed by the publisher.
