# Supplementary material for: Vaginal microbiota and HPV clearance: A longitudinal study
Source: Front Oncol. 2022 Oct 24;12:955150. doi: 10.3389/fonc.2022.955150 (PMC9639776; doi:10.3389/fonc.2022.955150)
Supplement: Supplementary file 1 [file DataSheet_1.docx]

Supplementary Material













**Supplementary Figure 1. Heatmap of the abundance of the 20 most abundant amplicon sequence variants (ASVs) or genus in the vaginal communities of enrolled women sampled at baseline.**

(A-E) Clustering on the abundance profiles of individual samples using the partitioning around medoids algorithm identified five community state types (CSTs) on ASVs levels. (F, G) CSTs clustered on genus level data.

**Supplementary Table 1. Pairwise comparisons of 12-month clearance rates for HR.HPV subtypes**

| Compare pairs | *P* value | Adjusted *P* value |
| --- | --- | --- |
| HPV16 vs HPV52 | 0.860 | 1.000 |
| HPV16 vs HPV58 | 0.947 | 1.000 |
| HPV52 vs HPV58 | 1.000 | 1.000 |

Note: HPV, human papillomavirus; P-values were adjusted using the Benjamini-Hochberg method

**Supplementary Table 2. Association between Shannon index of VM and patients’ characteristics**

| Characteristics | Univariable Model | |  | Multivariable Model1 | |  | Multivariable Model2 | |  | Multivariable Model3 | |
| --- | --- | --- | --- | --- | --- | --- | --- | --- | --- | --- | --- |
|  | Estimate | P value |  | Estimate | P value |  | Estimate | P value |  | Estimate | P value |
| **Age** |  |  |  |  |  |  |  |  |  |  |  |
| <50 year | Ref. |  |  |  |  |  | Ref. |  |  | Ref. |  |
| ≥50 year | 0.08 | 0.793 |  | 0.08 | 0.800 |  | -0.09 | 0.733 |  | -0.15 | 0.587 |
| **HPV type** |  |  |  |  |  |  |  |  |  |  |  |
| 16 | Ref. |  |  |  |  |  | Ref. |  |  | Ref. |  |
| 52 | -0.23 | 0.420 |  | -0.27 | 0.362 |  | -0.33 | 0.190 |  | -0.07 | 0.810 |
| 58 | -0.22 | 0.490 |  | -0.28 | 0.398 |  | -0.23 | 0.419 |  | -0.09 | 0.762 |
| **Diagnosis** |  |  |  |  |  |  |  |  |  |  |  |
| HPV+/LSIL | Ref. |  |  |  |  |  | Ref. |  |  | Ref. |  |
| HSIL | -0.11 | 0.693 |  | -0.17 | 0.562 |  | -0.03 | 0.911 |  | -0.02 | 0.935 |
| **CST** |  |  |  |  |  |  |  |  |  |  |  |
| I | Ref. |  |  |  |  |  | Ref. |  |  |  |  |
| II | 0.34 | 0.313 |  |  |  |  | 0.37 | 0.297 |  |  |  |
| III | 0.09 | 0.811 |  |  |  |  | 0.09 | 0.81 |  |  |  |
| IV | 1.56 | **<0.001** |  |  |  |  | 1.62 | **<0.001** |  |  |  |
| V | 1.21 | **0.001** |  |  |  |  | 1.22 | **0.002** |  |  |  |
| **CST_genus** |  |  |  |  |  |  |  |  |  |  |  |
| 1 | Ref. |  |  |  |  |  |  |  |  | Ref. |  |
| 2 | 0.96 | **<0.001** |  |  |  |  |  |  |  | 0.97 | **<0.001** |

Note: HPV, human papillomavirus; LSIL, low-grade squamous intraepithelial lesion; HSIL, high-grade squamous intraepithelial lesion; CSTs, community state types. HSIL patients received surgical resection, and LSIL/HPV+ patients underwent non-surgical treatment. Linear regression was conducted.

**Supplementary Table 3.** **Association between Chao1 index of VM and patients’ characteristics**

| Characteristics | Univariable Model | |  | Multivariable Model1 | |  | Multivariable Model2 | |  | Multivariable Model3 | |
| --- | --- | --- | --- | --- | --- | --- | --- | --- | --- | --- | --- |
|  | Estimate | P value |  | Estimate | P value |  | Estimate | P value |  | Estimate | P value |
| **Age** |  |  |  |  |  |  |  |  |  |  |  |
| <50 year | Ref. |  |  |  |  |  | Ref. |  |  | Ref. |  |
| ≥50 year | -0.85 | 0.921 |  | 1.29 | 0.882 |  | -0.75 | 0.923 |  | -4.74 | 0.560 |
| **HPV type** |  |  |  |  |  |  |  |  |  |  |  |
| 16 | Ref. |  |  |  |  |  | Ref. |  |  | Ref. |  |
| 52 | -16.96 | **0.038** |  | -16.55 | **0.05** |  | -18.24 | **0.015** |  | -11.22 | 0.154 |
| 58 | -11.95 | 0.174 |  | -11.37 | 0.22 |  | -8.15 | 0.322 |  | -6.52 | 0.449 |
| **Diagnosis** |  |  |  |  |  |  |  |  |  |  |  |
| HPV+/LSIL | Ref. |  |  |  |  |  | Ref. |  |  | Ref. |  |
| HSIL | 5.670 | 0.459 |  | 2.44 | 0.761 |  | 8.01 | 0.270 |  | 6.18 | 0.408 |
| **CST** |  |  |  | — | — |  |  |  |  | — | — |
| I | Ref. |  |  |  |  |  | Ref. |  |  |  |  |
| II | 1.47 | 0.886 |  |  |  |  | 2.19 | 0.831 |  |  |  |
| III | 6.01 | 0.584 |  |  |  |  | 8.51 | 0.433 |  |  |  |
| IV | 36.55 | **0.002** |  |  |  |  | 41.64 | **0.001** |  |  |  |
| V | 27.65 | **0.015** |  |  |  |  | 28.92 | **0.011** |  |  |  |
| **CST_genus** |  |  |  | — | — |  | — | — |  |  |  |
| 1 | Ref. |  |  |  |  |  |  |  |  | Ref. |  |
| 2 | 25 | **<0.001** |  |  |  |  |  |  |  | 25.17 | **0.001** |

Note: HPV, human papillomavirus; LSIL, low-grade squamous intraepithelial lesion; HSIL, high-grade squamous intraepithelial lesion; CSTs, community state types. HSIL patients received surgical resection, and LSIL/HPV+ patients underwent non-surgical treatment.





**Supplementary Figure 2.** **Differences in vaginal microbial β-diversity (Bray-Curtis distance) between community state types of enrolled patients.**

Notes: CSTs, community state types. CSTs were clustered on amplicon sequence variants data.

**Supplementary Table 4. Permutational analysis of variance (PERMANOVA) for** **the association between patients’ characteristics and the interpersonal distance (Bray-Curtis) of microbial composition**

| Characteristics | Univariable Model | |  | Multivariable Model | |
| --- | --- | --- | --- | --- | --- |
|  | R^2^(%) | *P* value |  | R^2^(%) | *P* value |
| age | 2.06 | 0.169 |  | 2.06 | 0.156 |
| HPV type | 3.12 | 0.333 |  | 3.09 | 0.305 |
| Diagnosis | 1.6 | 0.300 |  | 2.05 | 0.163 |
| CST | 53.78 | **0.001** |  |  |  |
| CST_genus | 13.46 | **0.001** |  |  |  |

Note： HPV, human papillomavirus; CSTs, community state types clustered on amplicon sequence variants data; CST_genus, community state types clustered on genus level.

**Supplementary Table 5. PERMANOVA result of pairwise comparisons of the microbial community composition between samples**

| Compare pairs | R^2^(%) | *P* value | *P*.adjusted |
| --- | --- | --- | --- |
| HPV58 vs HPV16 | 3.13 | 0.171 | 0.513 |
| HPV58 vs HPV52 | 1.63 | 0.651 | 0.651 |
| HPV16 vs HPV52 | 1.67 | 0.54 | 0.651 |

Note: *P*-values were adjusted using the Benjamini-Hochberg method.

**Supplementary Table 6.** **Association between VM and HPV clearance according to disease diagnosis/treatment**

| Strata | Characteristic | HPV-cleared | HPV-uncleared | OR | 95% CI | *P* value |
| --- | --- | --- | --- | --- | --- | --- |
| **HPV+/LSIL** | CST I | 2 (7.1%) | 3 (13.0%) | — | — |  |
|  | CST II | 8 (28.6%) | 6 (26.1%) | 0.50 | 0.05, 3.96 | 0.513 |
|  | CST III | 4 (14.3%) | 8 (34.8%) | 1.33 | 0.13, 11.8 | 0.794 |
|  | CST IV | 8 (28.6%) | 2 (8.7%) | 0.17 | 0.01, 1.63 | 0.138 |
|  | CST V | 6 (21.4%) | 4 (17.4%) | 0.44 | 0.04, 3.91 | 0.468 |
|  |  |  |  |  |  |  |
| **HSIL** | CST I | 4 (23.5%) | 1 (20.0%) | — | — |  |
|  | CST II | 7 (41.2%) | 1 (20.0%) | 0.57 | 0.02, 17.2 | 0.718 |
|  | CST III | 2 (11.8%) | 1 (20.0%) | 2.00 | 0.06, 73.7 | 0.676 |
|  | CST IV | 2 (11.8%) | 0 (0%) | 0.00 | 0.00, NA | 0.995 |
|  | CST V | 2 (11.8%) | 2 (40.0%) | 4.00 | 0.23, 126 | 0.355 |
|  |  |  |  |  |  |  |
| **HPV+/LSIL** | CST_genus 1 | 15 (53.6%) | 15 (65.2%) | — | — |  |
|  | CST_genus 2 | 13 (46.4%) | 8 (34.8%) | 0.62 | 0.19, 1.90 | 0.402 |
|  |  |  |  |  |  |  |
| **HSIL** | CST_genus 1 | 13 (76.5%) | 3 (60.0%) | — | — |  |
|  | CST_genus 2 | 4 (23.5%) | 2 (40.0%) | 2.17 | 0.23, 18.5 | 0.473 |
|  |  |  |  |  |  |  |
| **HPV+/LSIL** | Shannon(low) | 19 (67.9%) | 18 (78.3%) | — | — |  |
|  | Shannon(high) | 9 (32.1%) | 5 (21.7%) | 0.59 | 0.15, 2.04 | 0.410 |
|  |  |  |  |  |  |  |
| **HSIL** | Shannon(low) | 14 (82.4%) | 4 (80.0%) | — | — |  |
|  | Shannon(high) | 3 (17.6%) | 1 (20.0%) | 1.17 | 0.05, 12.5 | 0.905 |
|  |  |  |  |  |  |  |
| **HPV+/LSIL** | Chao1(low) | 19 (67.9%) | 21 (91.3%) | — | — |  |
|  | Chao1(high) | 9 (32.1%) | 2 (8.7%) | 0.20 | 0.03, 0.90 | 0.057 |
|  |  |  |  |  |  |  |
| **HSIL** | Chao1(low) | 11 (64.7%) | 3 (60.0%) | — | — |  |
|  | Chao1(high) | 6 (35.3%) | 2 (40.0%) | 1.22 | 0.13, 9.56 | 0.848 |

Note: Shannon index and Chao1 index were was categorized by 75th quartile scores.

HPV, human papillomavirus; LSIL, low-grade squamous intraepithelial lesion; HSIL, high-grade squamous intraepithelial lesion; CSTs, community state types. HSIL patients received surgical resection, and LSIL/HPV+ patients underwent non-surgical treatment.

**Supplementary Table 7. Association between VM and HPV clearance according to HPV subtypes.**

| Strata | Characteristic | HPV-cleared | HPV-uncleared | OR | 95% CI | *P* value |
| --- | --- | --- | --- | --- | --- | --- |
| HPV16 | CST I | 3 (17.6%) | 1 (11.1%) | — | — |  |
|  | CST II | 4 (23.5%) | 3 (33.3%) | 2.25 | 0.17, 59.4 | 0.558 |
|  | CST III | 2 (11.8%) | 4 (44.4%) | 6.00 | 0.43, 178 | 0.214 |
|  | CST IV | 3 (17.6%) | 0 (0%) | 0.00 | 0, NA | 0.994 |
|  | CST V | 5 (29.4%) | 1 (11.1%) | 0.60 | 0.02, 19.2 | 0.748 |
|  |  |  |  |  |  |  |
| HPV52 | CST I | 2 (12.5%) | 2 (18.2%) | — | — |  |
|  | CST II | 5 (31.3%) | 1 (9.1%) | 0.20 | 0.01, 3.28 | 0.278 |
|  | CST III | 3 (18.8%) | 3 (27.3%) | 1.00 | 0.07, 13.8 | >0.999 |
|  | CST IV | 4 (25.0%) | 2 (18.2%) | 0.50 | 0.03, 6.96 | 0.600 |
|  | CST V | 2 (12.5%) | 3 (27.3%) | 1.50 | 0.10, 24.8 | 0.765 |
|  |  |  |  |  |  |  |
| HPV58 | CST I | 1 (8.3%) | 1 (12.5%) | — | — |  |
|  | CST II | 6 (50.0%) | 3 (37.5%) | 0.50 | 0.02, 15.7 | 0.661 |
|  | CST III | 1 (8.3%) | 2 (25.0%) | 2.00 | 0.04, 120 | 0.711 |
|  | CST IV | 3 (25.0%) | 0 (0%) | 0.00 | 0, NA | 0.996 |
|  | CST V | 1 (8.3%) | 2 (25.0%) | 2.00 | 0.04, 120 | 0.711 |
|  |  |  |  |  |  |  |
| HPV16 | CST_genus 1 | 8 (47.1%) | 6 (66.7%) | — | — |  |
|  | CST_genus 2 | 9 (52.9%) | 3 (33.3%) | 0.44 | 0.07, 2.29 | 0.345 |
|  |  |  |  |  |  |  |
| HPV52 | CST_genus 1 | 12 (75.0%) | 7 (63.6%) | — | — |  |
|  | CST_genus 2 | 4 (25.0%) | 4 (36.4%) | 1.71 | 0.31, 9.55 | 0.527 |
|  |  |  |  |  |  |  |
| HPV58 | CST_genus 1 | 8 (66.7%) | 5 (62.5%) | — | — |  |
|  | CST_genus 2 | 4 (33.3%) | 3 (37.5%) | 1.20 | 0.17, 7.97 | 0.848 |
|  |  |  |  |  |  |  |
| HPV16 | Shannon(low) | 11 (64.7%) | 7 (77.8%) | — | — |  |
|  | Shannon(high) | 6 (35.3%) | 2 (22.2%) | 0.52 | 0.06, 3.08 | 0.496 |
|  |  |  |  |  |  |  |
| HPV52 | Shannon(low) | 12 (75.0%) | 11 (100%) | — | — |  |
|  | Shannon(high) | 4 (25.0%) | 0 (0%) | 0.00 | 0.00, NA | 0.995 |
|  |  |  |  |  |  |  |
| HPV58 | Shannon(low) | 10 (83.3%) | 4 (50.0%) | — | — |  |
|  | Shannon(high) | 2 (16.7%) | 4 (50.0%) | 5.00 | 0.69, 48.6 | 0.125 |
|  |  |  |  |  |  |  |
| HPV16 | Chao1(low) | 9 (52.9%) | 7 (77.8%) | — | — |  |
|  | Chao1(high) | 8 (47.1%) | 2 (22.2%) | 0.32 | 0.04, 1.81 | 0.226 |
|  |  |  |  |  |  |  |
| HPV52 | Chao1(low) | 12 (75.0%) | 11 (100%) | — | — |  |
|  | Chao1(high) | 4 (25.0%) | 0 (0%) | 0.00 | 0.00, NA | 0.995 |
|  |  |  |  |  |  |  |
| HPV58 | Chao1(low) | 9 (75.0%) | 6 (75.0%) | — | — |  |
|  | Chao1(high) | 3 (25.0%) | 2 (25.0%) | 1.00 | 0.11, 7.95 | >0.999 |

Note: All models were adjusted for disease diagnosis at baseline. Shannon index and Chao1 index were categorized by 75th quartile scores.

HPV, human papillomavirus; HSIL, high-grade squamous intraepithelial lesion; LSIL, low-grade squamous intraepithelial lesion; CSTs, community state types. HSIL patients received surgical resection, and LSIL/HPV+ patients underwent non-surgical treatment.
